# Characterization of Microalgae Biomass-Based Composites Obtained through Rotational Molding

**DOI:** 10.3390/polym16131807

**Published:** 2024-06-26

**Authors:** Sara Díaz, Francisco Romero, Luis Suárez, Raúl Ríos, Monserrat Alemán, Marianna Venuleo, Zaida Ortega

**Affiliations:** 1 Departamento de Ingeniería de Procesos, Universidad de Las Palmas de Gran Canaria, Edificio de Fabricación Integrada, Parque Científico—Tecnológico de la ULPGC, Campus Universitario de Tafira Baja, 35017 Las Palmas de Gran Canaria, Las Palmas, Spain; francisco.romero@ulpgc.es (F.R.); luis.suarez@ulpgc.es (L.S.); 2 Instituto Tecnológico de Canarias (ITC), Playa de Pozo Izquierdo, s/n, 35119 Santa Lucía, Las Palmas, Spain; rjrios@itccanarias.org (R.R.); maleman@itccanarias.org (M.A.); mvenuleo@itccanarias.org (M.V.)

**Keywords:** biocomposite, microalgae, Spirulina, rotomolding, antioxidant

## Abstract

The wide range of applications and the numerous advantages of plastics have led to their excessive use, with subsequent damage to ecosystems. As an environmentally friendly alternative, biocomposites have gained much attention, and microalgae have become a potential source for their production. In this study, the use of washed and unwashed Spirulina in polyethylene-based composites has been evaluated as a way to prevent the thermooxidation of polyethylene, while at the same time, reducing the amount of virgin plastic used. Biocomposites were produced by rotomolding, testing different biomass contents and determining their mechanical and thermal performances as well as their water uptake level. Composites with up to 15% of biomass (by weight), a particularly high ratio for rotomolding, were satisfactorily produced. Using 5% of both biomasses did not significantly modify the behavior when compared with the neat PE samples’ properties. For higher loadings, the use of non-washed biomass allowed us to obtain better properties, with added benefits related to using an unwashed biomass (less water consumption, lower costs and fewer environmental impacts). On the other hand, this study showed a promising beneficial effect on the thermooxidative resistance of composites, as the oxidation induction times were notably increased with biomass addition.

## 1. Introduction

Due to their light weight, cost-effectiveness and durability, among other advantages, plastic materials are intensively used nowadays in many applications, including agricultural films, packaging, disposal consumer items, health, construction, etc. [1]. On the other hand, almost 50% of these plastic materials, mainly non-degradable, are consumed for single-use disposable applications [2], generating serious environmental problems. Sensitiveness to the damage caused by these materials, including the emission of greenhouse gases during the production and accumulation of residues in the ecosystem, has increased and has resulted in an increased awareness of the search of more eco-friendly materials, developing a series of biocomposites as an environmentally compatible solution [3]. 

The term biocomposite includes materials prepared using a biopolymer with an organic or biomass-based filler, or composites made of an oil-based polymer with biobased fillers. Both approaches have attracted a considerable number of researchers and scholars worldwide and are currently used in different applications, mainly in the packaging, automotive, furniture and construction industries [4]. Concretely, the inclusion of natural fillers in the thermoplastic matrix has become one of the biggest areas of research in recent years, as they offer different advantages, such as cost and weight reductions, reduce the dependence on fossil resources, and reduce the wastes related to non-degradable composites in the environment [5].

On the other hand, rotational molding is an extensively used manufacturing process that allows one to obtain hollow parts, with a high surface quality, good thickness distribution and good mechanical properties [6]. It is characterized by a low or null shear rate and low costs for tooling and equipment [7]. However, the process presents some disadvantages, such as its relatively long cycle time, particularly if compared to injection molding, which is related to a low energy efficiency, together with the narrow range of materials suitable to be processed using this technology [8]. Regarding the use of natural fillers in the production of composites using rotomolding technology, just a few studies have been conducted due to the high sensitivity of rotational molding to the introduction of foreign materials, which results in the worsening of the mechanical properties of the rotomolded items, mainly the impact resistance [6]. The authors of this study have extensively contributed to the published literature in this field, incorporating several types of filler and reinforcement into rotomolded products [9,10]. Some examples of natural fillers used in rotomolding include lignocellulosic fibers, such as wheat bran [7], abaca, banana or agave [11,12] fibers, or black tea waste [13], although generally not exceeding 10% by weight [14]. In some studies, this amount has been increased using a specific particle size distribution for the filler or through the use of compatibilizers [14]. Regarding the microalgae biomass, the raw material chosen as a filler in the present study, although considered as a potential source for biocomposite production, has not been used in rotational molding to the best of the authors’ knowledge. However, some studies have obtained composites with microalgae using compression molding [15,16], solvents (for bio-films production) [15], and injection [17,18,19]. Therefore, this is the first work studying the incorporation of this biomass using this processing technology. Only in a preliminary previous study by the authors of this study, *Tetraselmis* biomass was incorporated in the rotomolding process, showing the importance of choosing a suitable biomass and appropriate processing conditions in order to avoid biomass degradation [20].

Mass cultures of microalgae are regarded as a promising alternative to traditional terrestrial crops for the production of food, feed and biofuels, among other products. Outdoor microalgal cultures present several advantages, such as fast growth with high yields, when performed in non-arable land and with the use of low-quality (and low cost) water, such as seawater, wastewater or brackish water; furthermore, exhaust gases with CO_2_ can be used as a carbon source during cultivation [21]. Moreover, microalgae can be used in waste water treatment, as they are able to absorb excess nutrients from waste water [22]. In addition, microalgae play an important role in controlling CO_2_ concentrations in the atmosphere [23]. The blue economy arises as a pillar for the achievement of sustainable development goals and to meet the regulations in terms of research and development. This sector contributes to about 1.5% of the European GDP and to over 2% of total EU employment [24]. Blue economy sectors contribute to about 1.5% of the EU-27 GDP, with approximately 4.5 million directly related jobs, which means that 2.3% of EU-27 total employment is related to this sector. Emerging technologies, such as the production of macro- and microalgae, are creating new markets and jobs and contributing to the diversification of the economy.

The studies found on the production of biocomposites from microalgae biomass can be separated into two main categories: the production of composites by combining microalgae with different polymers, either bio- or petroleum-based [16,25,26,27,28,29], and those based on the cultivation of biopolymers such as polyhydroxybutyrates (PHBs) [30,31,32]. Regarding this last group, further research into bioplastics obtaining is still needed to overcome the economic feasibility issues that are hindering the broad use of such materials in the industrial sector [33]. 

In this study, the first of the two previously commented options, in which the biomass was combined with a polymer, was explored as a way to produce parts with improved functionality, i.e., higher resistance to thermooxidation, which is an important phenomenon within the rotational molding industry. Among the wide variety of microalgae available, *Chlorella* and Spirulina (cyanobacteria) are the most commonly studied ones in the composites sector [34,35]. Spirulina (a well-studied cyanobacterium with a well-defined production method, as it is commonly produced on a large scale for nutraceutical applications [36]) was chosen as the raw material for the present study. Spirulina is recognized as a source of protein, with up to 70% protein content [37]. This high protein content, as well as its small size, have made Spirulina suitable for plastic conversion [36]. In addition, in the present study, the biomass was used in two different formats, after washing with potable water for salt removal, and it was directly used without previous washing; this aspect, to the best of our knowledge, has not been evaluated in the literature. The use of non-washed biomass can be a more environmentally sustainable and cost-effective option, as clean water is not used for this purpose, but also the presence of salt could have a positive effect on the mechanical properties of the composites, as has also been found for bioplastics derived from this source [38]. In their research work, Zhang et al. studied the utilization of Spirulina sp. residue generated after phycocyanin extraction in the production of bioplastic films [38]. This residue contains large amounts of inorganic salts, such as calcium chloride and disodium hydrogen phosphate, that could enhance the elongation or tensile strength of the bioplastic. However, no studies about the effect of these salts on rotomolded PE have been found in the literature to date.

Polyethylene (PE) and polypropylene (PP) are the most commonly used to date to produce blended bioplastics from microalgae [17,25,27,28]. In the case of Spirulina, apart from these two polymers, its biomass has been combined with UHMW-PE [29] and PBS [22]. PVC [26], PU [17,39], PVA [4,16,34,40] and PCL [41] have been also used in combination with other microalgae for different possible applications. In the present study, PE was used as a matrix. Over 85% of the parts produced by rotational molding worldwide are made from PE [42] due to its low melting point, low cost and good thermal stability. The use of PE allows for using low temperatures, thus reducing the risk of biomass thermal degradation. 

The incorporation of microalgae biomass in rotomolding not only would have an environmental benefit, as part of the polymer is replaced by material that comes from a renewable resource, but it could have a special interest due to the high content of antioxidants [43]. Over 50 compounds with high biological activity have been obtained from Spirulina [37]. The incorporation of antioxidants to prevent the thermooxidation of polymers processed by rotational molding is essential due to the long processing times and high temperatures needed in this process. The interest in natural antioxidants has grown recently, as they can act as good stabilizers for polyolefins [44]. They have been introduced as pure antioxidant compounds isolated from fruits and plants or as extracts from plants or herbs which are rich in antioxidants. In addition, the direct use of fruit and plant wastes has been evaluated [13]. For example, adding coffee grounds or by-products into PE increases the oxidation induction time (OIT) [45]. Similar results were obtained for HDPE with cocoa husks [46], or by incorporating black tea waste in rotomolded polyethylene [13]. In relation to microalgae, although not specifically in rotomolding, recent studies have been conducted in which the stabilization potential of microalgae in biocomposites with simple polymers (such as PE and PP) has been demonstrated. A recent report concluded that the use of microalgae (*Chlorella vulgaris* and *Spirulina platensis*) in PP was able to prevent the degradation of PP, primarily due to the polyphenol content in microalgae [47]. On the other hand, Tafreshi et al. also reported that polyethylene glycol (PEG) combined with *Chlorella vulgaris* significantly ameliorated the stress caused by gamma irradiation [48].

In this paper, the addition of Spirulina biomass in polyethylene-based composites to improve the environmental behavior of rotomolded items, while at the same time providing interesting features such as stabilization, will be discussed. Biocomposites were produced by rotational molding, making this the first study, to the best of our knowledge, in which microalgae biomass is used in this type of molding process. A comparison between the use of washed and non-washed Spirulina biomasses on the properties of the parts was also conducted, which is an aspect that has not been studied in the literature and that has an impact on the economy and sustainability of the process. The effect of the biomass content ranging from 5% to 15% on the mechanical, thermal and water-absorption properties of biocomposites was investigated. Composites with decent mechanical properties and improved thermooxidative resistance were obtained.

## 2. Materials and Methods

### 2.1. Materials

#### 2.1.1. Strain Isolation and Outdoor Cultivation

*A. platensis* (Spirulina) strain BEA 1257B was isolated by the Spanish Bank of Algae (BEA, Telde, Gran Canaria, Spain) from the reservoir of Los Molinos, Dam-Betancuria Rural Park, Fuerteventura (Spain, 28°30026″ N, 14°01047″ W), in 2014. Optical microscopy was used for morphological identification, and 16S gene sequencing (GenBank MT426015) confirmed the identity of the species. At the Canarian Institute of Technology (ITC), the culture was scaled up from a culture chamber (8 L inoculum) to outdoor raceway reactors of increasing volumes (from 250 L to 1600 L) up to a 80 m^2^ raceway reactor; here, *A. platensis* was grown semi-continuously in 100% seawater with a culture medium modified by Guidi et al. [21]. 

#### 2.1.2. Biomass Harvesting and Processing 

**Figure 1:**
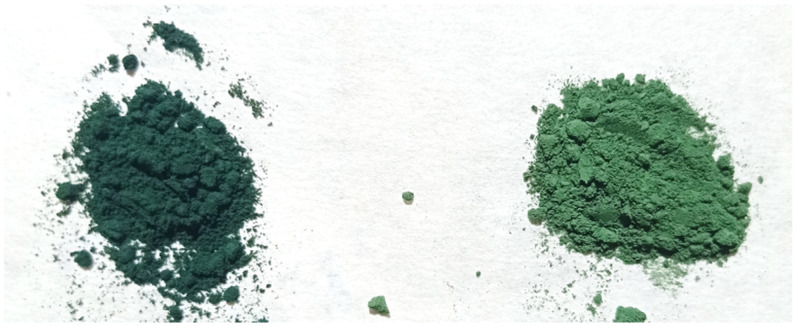
Spirulina (**left**) and non-washed Spirulina (**right**) biomasses.

The harvesting of the biomass was performed at the ITC through an industrial circular vibrating screen (Filtra^®^ FTI-0800, Ø 800 mm, filtration area 0.5 m^2^; Filtra Vibración, Barcelona, Spain) using a 15 µm stainless steel net. The algal slurry obtained after harvesting was either washed (5 L freshwater kg^−1^ biomass) and manually pressed through a 30 µm nylon net before freezing at −20 °C, or frozen directly without further processing. Washing and pressing allowed us to obtain a reduction in biomass salt content (as suggested by Guidi et al. [21]). Afterwards, all biomasses were dehydrated by spray-drying (Model L-12, Ohkawara Kakohki Co., Ltd., Yokohama, Japan), obtaining a dried washed/pressed biomass (thereinafter Sp) and a dried unwashed/non-pressed biomass (thereinafter Sp.N.W). Both biomasses can be observed in Figure 1. The spray-dried *A. platensis* biomass had a water content of 7.5% (*w*/*w*). While the relative organic composition (59.8% proteins, 5.1% lipids and 34.9% total carbohydrates; ash-free dry weight) was not affected by the washing/pressing protocol, the quantity of inorganics was significantly lower in the Sp biomass (23.3%, *w*/*w*) compared to that in the Sp.N.W biomass (30.8%, *w*/*w*).

### 2.2. Composite Processing

The polymer matrix (polyethylene (PE) Revolve N-461 from Matrix Polymers, Northmapton, UK) and the microalgae biomass (Sp or Sp.N.W) were dry-blended at the corresponding ratio. The PE and microalgae biomasses were previously dried in an oven at 60 °C for 24 h and 6 h, respectively. Biomass contents of 5, 10 and 15% (by weight) were used. The samples obtained and included in Table 1 were named with the letter R, corresponding to the rotomolding process, the matrix used (PE) followed by the percentage of biomass (5, 10 or 15%) and the type of biomass (Sp: Spirulina; Sp.N.W: non-washed Spirulina).

The mixed materials (242 g) were introduced in an aluminum cube-shaped mold obtaining parts with 3–4 mm thickness. A lab-made rotomolding machine (clamshell type with air convection heating/cooling system) described in a previous work [6] was used. The air temperature inside the mold was monitored with a thermocouple; the heating step finished when the temperature inside the mold reached the desired temperature (fixed at 168 °C after some previous tests). 

### 2.3. Characterization

#### 2.3.1. Mechanical Characterization

For mechanical characterization, test bars were die-cut from the cube sides following standard protocols. Flexural and tensile tests were performed following the recommendations established in UNE-EN ISO 178:2011 [49] and UNE-EN ISO 527-2:2012 [50], respectively, using an universal testing machine from Dongguan Liyi Test Equipment (model LY-1065, Dongguan, China); test rates of 2 mm/min for modulus determination and 10 mm/min for ultimate strength determination were used. Impact tests were performed with Dongguan Liyi Test Equipment (model LY-XJJD 50, China) apparatus following the UNE-EN ISO 180:2001/A2:2013 standard [51] using a 5.5 J pendulum and impact rate of 3.5 m/s. Six samples were tested for each property.

#### 2.3.2. Thermal Behavior

The differential scanning calorimetry (DSC) of both biomasses and composite materials was conducted using PerkinElmer DSC 4000 apparatus (PerkinElmer, Waltham, MA, USA). The samples (9–12 mg) were introduced in aluminum pans and placed in the DSC cell. First, the samples were heated from 30 °C to 200 °C under a nitrogen atmosphere using a heating rate of 10 °C/min. The samples were then cooled down at the same rate and heated again to 200 °C under the same conditions. The melting temperature for both heating rounds (T_m1_ and T_m2_) and crystallization temperature (T_c_) were determined. Cold crystallization was not observed for any of the samples. Melting and crystallization enthalpies (ΔH_m1_, ΔH_m2_, and ΔH_c_) were used to determine the degree of crystallization using the following expression (Equation (1)):

χ = ΔH_m2_/(ΔH_0_·(1 − m_Sp_)) × 100 (1)

where ΔH_0_ is the enthalpy for 100% crystalline sample (293 J/g for PE [52]), and m_Sp_ is the mass fraction of the microalgae.

The oxidation induction time (OIT) of the composite materials was also determined by DSC following the UNE-EN ISO 11357-6:2018 standard [53]. In short, the 10 mg sample was placed in an open aluminum pan and heated from 30 to 210 °C with a heating rate of 20 °C/min in nitrogen, and then kept at 210 °C for 5 min also under a nitrogen atmosphere. The gas was then switched to air (at a flow rate of 50 mL/min). The OIT was determined as the time to the onset of oxidation (considering that the initial heating time under N_2_ was subtracted).

The thermal decomposition temperatures of the biomass and composites samples were determined using a thermogravimetric analyzer from Perkin Elmer (TGA 4000 device, Waltham, MA, USA). Heating (from 30 to 600 °C at 10 °C/min) was conducted under a nitrogen atmosphere (40 mL/min) for oxygen-free pyrolysis. Samples of nominally 5 mg were used in the analysis. A derivative thermogravimetric (DTG) curve was also calculated using TG. 

#### 2.3.3. Fourier-Transform Infrared Spectroscopy (FTIR)

A PerkinElmer Spectrum Two spectrophotometer working in the attenuated total reflectance (ATR) mode was utilized to obtain the FTIR spectra of all the sample series. The device was settled to a resolution of 4 cm^−1^, accumulating 12 scans per samples in the range from 4000 to 500 cm^−1^.

#### 2.3.4. Microscopic Observations

An Olympus BX51 microscope (Olympus, Tokyo, Japan) was used in order to observe the original biomass and biomass distributions inside the matrix.

Scanning electron microscopy (SEM) analysis (Hitachi TM3030 tabletop (Hitachi, Tokyo, Japan) settled at an acceleration of 15 kV) was also performed on the samples after covering them with a thin layer of Pd/Pt (SC 760 apparatus from Quorum Technologies Ltd. (Ringmer, UK) under argon). The original biomasses, the surface of the rotomolded parts and the breaking section of the samples after tensile test were observed to determine biomass–matrix bonding.

#### 2.3.5. Water Absorption

Water absorption tests were carried out according to UNE-EN ISO 62:2008 [54] for a total period of 2 months by immersing the samples in deionized water and weighing them periodically. Three replicates per sample were assessed. The samples were also used for the determination of water soluble matter loss and the correction of water absorption calculations. Water absorption (W) was calculated using the following equation:

W(%) = ((W_t_ − W_0_) + (W_0_ × Solu))/W_0_ × 100 (2)

where W_0_ is the initial mass of the sample, W_t_ the wet mass of the sample at t time, and Solu is the percentage of water soluble material, calculated as (W_0_ − W_td_)/W_0_, with W_td_ being the mass of the dried sample at t time.

### 2.4. Determination of the Total Phenolic Compounds 

Extractable polyphenols were obtained according to Pérez-Jiménez et al. [55]. A total of 1 g of biomass was extracted with 20 mL of methanol/water (50:50, *v*/*v*; pH 2) acidified with 2 N HCl for 1 h. After that, the sample was centrifuged, the supernatant recovered, and a second extraction was carried out using 20 mL of acetone/water (70:30, *v*/*v*) for 1 h. Both extracts were combined to quantify the total phenolic content (TPC) using the Folin–Ciocalteu colorimetric method described by Singleton et al. [56], and the results are expressed as gallic acid equivalents (GAEs) in mg per 100 g of dried material. For this assessment, two replicates were performed for each biomass.

### 2.5. Statistical Analysis 

All tests were replicated, and the results were calculated and are expressed as the mean ± the standard deviation. The results were analyzed by a multifactor statistical analysis of variance (Kruskal–Wallis test). To identify significant differences between groups, Tukey–Kramer tests were performed, settling the confidence level to 95%. Kruskal–Wallis and the multcompare function of MATLAB were used for statistical analysis.

## 3. Results

### 3.1. Biomass Characterization

**Figure 2:**
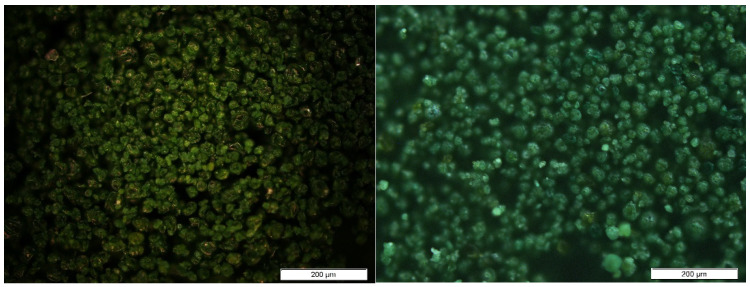
Pictures of Spirulina (**left**) and non-washed Spirulina (**right**) taken under optical microscope.

Figure 2 shows photographs of both the biomasses taken under an optical microscope. As shown, the biomasses exhibit a small particle size, generally under 50 µm, which can result in a homogeneous dispersion in the PE matrix.

**Figure 3:**
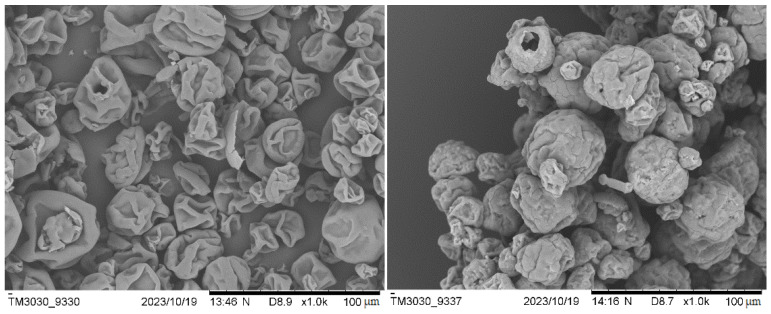
SEM images of Spirulina (**left**) and non-washed Spirulina (**right**).

SEM on the microalgal biomasses was performed in order to assess their size, shape and surface appearance. The images obtained for Sp and Sp.N.W are shown in Figure 3. As can be observed, the washing and pressing processes affected the biomass appearance. The non-washed biomass has a spherical shape with probably the presence of salts incrusted in its surface. Meanwhile, the washed Spirulina presents a more irregular shape. On the other hand, scanning electron microscopy with an Energy Dispersive X-ray (SEM-EDX) Analytical System allowed us to obtain the elemental composition of the surface (included in Table S1 in the Supplementary Materials). The Sp.N.W biomass, due to the presence of salt, presented higher concentrations of chlorine (47.50% for Sp.N.W vs. 20.97% for Sp) and sodium (22.80% for Sp.N.W vs. 9.42% for Sp).

**Figure 4:**
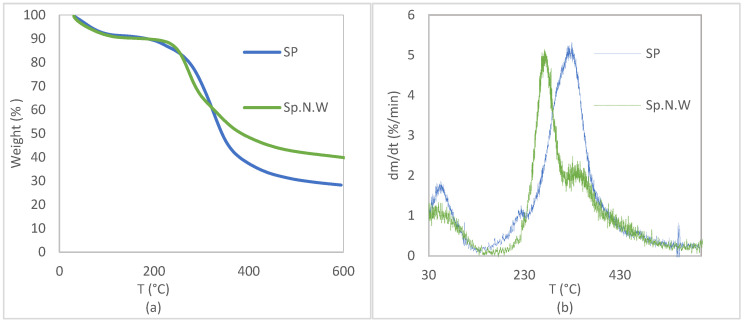
Thermogravimetric (TGA) (**a**) and derivative (DTG) (**b**) curves for Spirulina and non-washed Spirulina.

TGA and DTG curves of the Spirulina and non-washed Spirulina biomasses are represented in Figure 4. Given the repeatability of the results, in order to facilitate visualization, one of the replicates for each biomass is represented. Figure 4 shows that the thermal decomposition process of both biomasses was divided into three stages. The first one, between 30 and around 140 °C, mainly corresponded to the water dehydration process and also light volatile component evaporation. After that, both the biomasses showed two-step degradation, but at different temperatures, indicating that the presence of salt in the biomass alters its thermal behavior. In the Table 2, the weight loss obtained in the temperature range corresponding to each stage of decomposition is included for both the biomasses. 

The main peak temperatures in the DTG curves (330 °C and 274 °C for Sp and Sp.N.W, respectively) suggest protein decomposition, which is the main nutrient of the Spirulina biomass, and also the thermal degradation of lipids and carbohydrates, as reported by Jamilatun et al. [57]. Residual char levels of 27.95 ± 2.27% and 40.45 ± 0.51% were obtained for Sp and Sp.N.W, respectively. As expected, due to the presence of salt in Sp.N.W, the ashes value was considerably higher.

**Figure 5:**
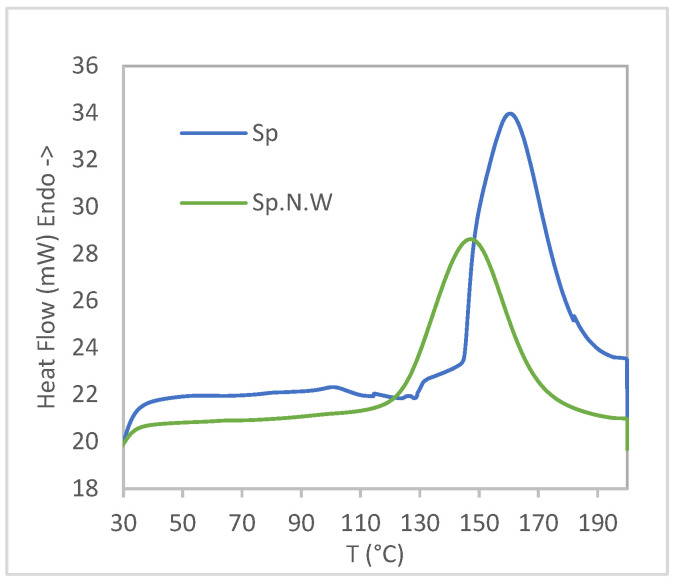
DSC curves for Spirulina and non-washed Spirulina samples for first heating.

Only the first heating stage during DSC is represented in Figure 5; the cooling and second heating stages did not present any thermal transition, suggesting a non-reversible process occurred during the first heating step. Both the biomasses showed a high peak that Zeller et al. attributed to protein denaturation [28]. For the non-washed Spirulina, this peak appears between 110 and 190 °C, with a maximum at 146.4 ± 1.9 °C. In the case of the washed Spirulina biomass, the curves obtained did not present a clean base line as happened with Sp.N.W, so some small thermal transitions could have occurred; the main peak took place in this case at 158.6 ± 4.7 °C. In contrast, Zeller et al. obtained a peak denaturation for Spirulina quite earlier, with a maximum at about 100 °C and ending at 170 °C, although in their case, they used a heating rate of 20 °C/min during DSC analysis, which could have affected the result [28]. 

Thus, based on the results obtained using both thermogravimetric analysis and differential scanning calorimetry, it can be concluded that both the biomasses present different thermal behaviors. Taking into consideration the results obtained during thermal analysis, a better processability for Sp.N.W might be expected, as protein denaturalization takes place before, and degradation starts slightly later. As exposed in the Materials and Methods section, a temperature of 168 °C was chosen for rotomolding the parts, ensuring the complete melting of the PE matrix. For the chosen temperature, about 10% weight loss was obtained for both the biomasses, corresponding mostly to water evaporation. With respect to the denaturation of the protein, the chosen temperature is located after the temperature of the peak obtained for both the biomasses. However, the complete denaturation of the protein was not obtained at this temperature; 79% and 91% protein denaturation (calculated from the percentage of the area under the curve) for Sp and Sp.N.W, respectively, were obtained.

**Figure 6:**
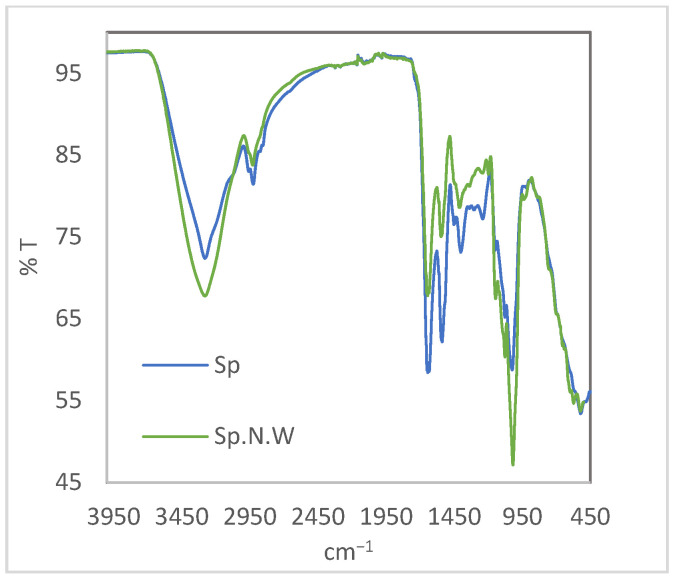
FTIR spectra for Spirulina and non-washed Spirulina biomasses.

On the other hand, although, due to the complexity of the spectra obtained for this kind of sample, determining the composition of biomasses using this technology is difficult, FTIR analysis was conducted in order to detect the possible differences in the composition of the biomasses. Figure 6 shows the FTIR spectra obtained for the Spirulina and non-washed Spirulina. As can be seen, both the biomasses presented a similar spectrum, with the presence of characteristic bands of the groups present in the main constituent of microalgae biomass (proteins, lipids and carbohydrates). In the case of Sp.N.W, some peaks were not as clear as those in the Sp spectra, which could be due to the presence of salt in the surface of the material.

The main peaks present in the spectra have been previously identified by other authors [58,59,60,61]. In some cases, it is difficult to assign a band to a specific compound (protein, lipid or carbohydrate) as they share regions of the spectra. For example, the peak at 3273 cm^−1^ corresponds to the stretching vibrations of O-H of polysaccharides, water molecules and proteins and the stretching of N-H (proteins). At the wavenumber range 2940–2853 cm^−1^, different vibrations of C-H can be observed [62]. The band at around 1634 cm^−1^ can be related with the presence of lipids (C=O ester absorption band), but also it can be attributed to the (C=O) stretching of amides from protein, the main constituent of Spirulina [63]. At 1542 cm^−1^, the amide II (proteins) band, which results from N-H bending and C-N stretching vibrations, was observed [58]. The bands between 1447 cm^−1^ and 1237 cm^−1^ can be also assigned to protein (the protein COO– side chain and amide III band) [58]. Finally, the bands observed in the range 1190 cm^−1^ and 950 cm^−1^ can be associated with the carbohydrate characteristic bands of C-O and C-C stretching [58].

### 3.2. Rotomolded Composites

#### 3.2.1. Visual Evaluation

**Figure 7:**
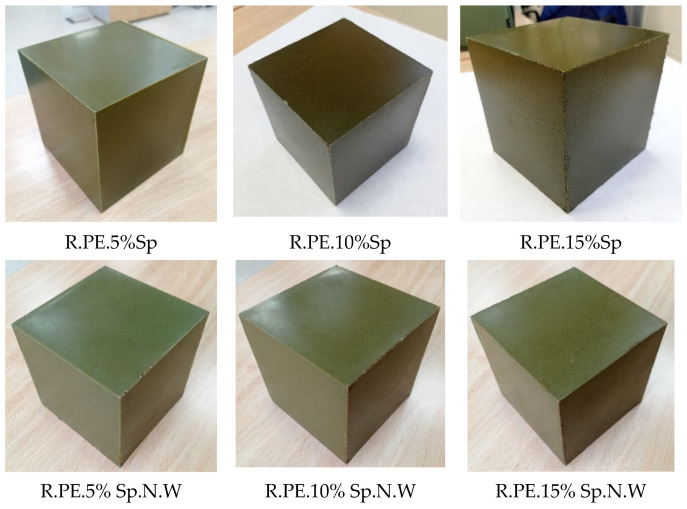

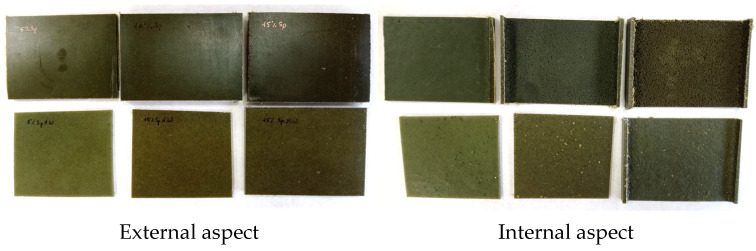
Pictures of rotomolded composites obtained with both biomasses.

The visual analysis of the rotomolded parts show a quite uniform green color for all the samples, as can be seen in Figure 7, indicating the proper distribution of the biomass into the matrix during the process. The external surfaces of the pieces are smooth, since they are in contact with the mold during the process, with the presence of very small bubbles that increased with the percentage of biomass incorporated. In the case of the Sp composites, these small bubbles resulted in some voids in the corners of the cube-shaped pieces, which were considerable for the ratio of 15%. In any case, these defects are mostly due to the small size of the oven in contrast with the mold size. However, in the case of the Sp.N.W composites, even for the highest percentage of biomass tested, an adequate surface finish was obtained. 

A rough appearance was observed for the internal surfaces of the cubes. This is a common characteristic of rotomolded parts, as during the process, the plastic particles melt and deposit in layers without a high pressure that controls material densification. A rougher inner surface with a higher presence of voids was observed for the higher loadings of biomass. As observed in the external surfaces, the presence of internal voids was also higher for the Sp composites. These differences in the amount of voids can be related to the difference in the bulk density of both the biomasses. In the case of the non-washed Spirulina, it presents a considerably higher bulk density, and so the same amount of material occupies a smaller volume, which could explain, in part, the difference observed between both the materials. Also, the different thermal behaviors may have influenced the inner appearance.

The density of the composite materials was calculated from the measurement of the dimensions and the weight of the samples and is included in Table 3. The lower density of the composites can be explained by the lower density of the biomass, which is translated into thicker sections of the rotomolded products. On the other hand, the incorporation of foreign materials into rotomolding is known to increase the porosity of the obtained items, leading to a reduction in density [12]. Since the biomass density values were not available, just the porosity of PE sample could be calculated (4.6%), which was found to be within an acceptable range. Due to the difference between the bulk densities of the biomasses, previously mentioned, the density of the Sp.N.W composites did not decrease as much as the density of the Sp composites. In any case, from visual inspection and microscopic observation, the presence of voids and bubbles was not significantly increased as a consequence of biomass incorporation. 

#### 3.2.2. Mechanical Characterization

**Figure 8:**
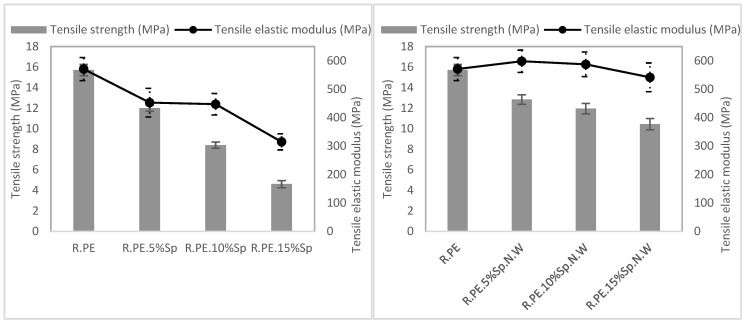
Tensile properties of washed Spirulina (**left**) and non-washed Spirulina (**right**) composite materials.

**Figure 9:**
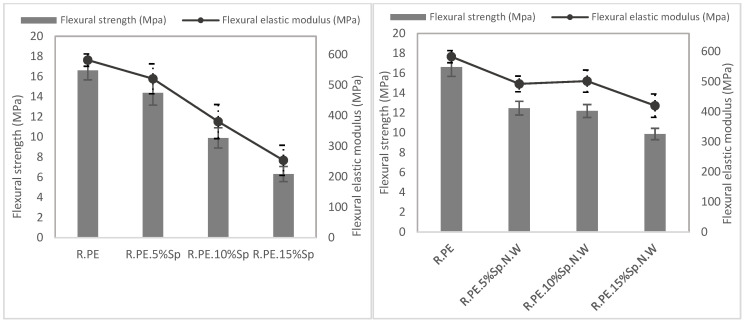
Flexural properties of washed Spirulina (**left**) and non-washed Spirulina (**right**) composite materials.

**Figure 10:**
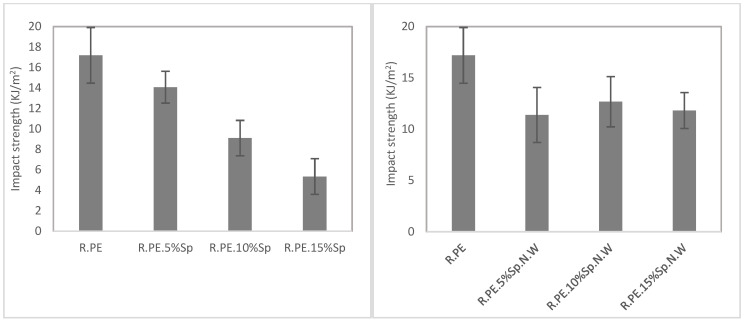
Impact properties of washed Spirulina (**left**) and non-washed Spirulina (**right**) composite materials.

Table 3 shows the results of the mechanical characterization of rotomolded composites obtained with both the biomasses (Sp and Sp.N.W). For better interpretation of the results presented in the table, the mechanical properties are also summarized in Figure 8, Figure 9 and Figure 10. In general, all the mechanical properties were affected (reduced) by the introduction of the filler when compared with those of the neat PE properties, although they were less affected in the case of the non-washed Spirulina.

The graphical representations of the results obtained for the Sp composites (graphs on the left in Figure 8, Figure 9 and Figure 10) showed that a linear (negative) relationship exists between the mechanical properties and the percentage of Spirulina. Excellent regression coefficients were obtained for all the properties, except for the tensile elastic modulus, as observed in Table 4. In the case of non-washed Spirulina (graphics on the right), this linear trend was not detected, although a common tendency was observed in almost all the properties. There is an initial deterioration of the properties for 5% of Sp.N.W, after which the properties do not drastically worsen. For example, compared with the neat PE, the tensile strength was reduced by 18% when 5 wt% of Sp.N.W was incorporated, while when the percentage of biomass was tripled (15%), the property was not affected by the same proportion (a reduction of 34% was obtained, which means 1/1.5). Regarding the tensile elastic modulus, it presented a different behavior than the other variables. There was no significant difference between the groups (*p*-value of 0.214), meaning its value was practically unaltered. The mechanical performance obtained for Spirulina–PE composites follows the trends observed generally for rotomolded composites, that is, an increase in tensile elastic modulus and a decrease in tensile strength. The literature shows that the nature and geometrical features of the filler introduced significantly affects the tensile and impact strength, with fibrous materials (with relatively high aspect ratio) providing higher tensile resistance, while the modulus tends to remain unchanged, or even increases [12,64]. The tensile strength usually is reduced as the filler results in a non-continuous plastic matrix, and the effort transmission worsens, while in the case of fibers (or rigid fillers), the elastic modulus is increased as a consequence of the stiffening provided by such fillers. For example, using 5% hemp fibers, Oliveira et al. reported a reduction in tensile strength, but in an increase in elastic modulus higher than 50% [15]; Hejna et al. determined a similar trend, i.e., a reduction in tensile strength and an increase in elastic modulus when they increased the ratio of filler used (gypsum dust in this case) [65]. 

As previously commented, one of the main limitations of rotational molding is the high sensitivity of the process to the introduction of foreign materials as a consequence of a lack of internal pressure, which generally result in the deterioration of the mechanical properties, mainly the impact strength [14]. In this case, the impact strength of the Sp composites was gradually reduced from a 20% (in comparison with that of neat PE) for 5% of biomass until achieving a 69% reduction in the property for the 15% composites. On the other hand, the composites with unwashed Spirulina show a different behavior, with an important reduction in the impact strength of about 34% for all the composites series, regardless of their filler ratio. The other studies found in the literature for composites based on a polyethylene matrix with natural fibers have obtained even larger drops for polyethylene composites. For example, using 5% banana and abaca fiber resulted in a reduction close to 90% in impact energy [11], Abhilash et al. found a similar behavior for LLDPE composites with 5% bamboo [66]; Torres et al. obtained reductions over 50% with respect to neat PE for sisal and cabuya composites at a maximum loading of 7.5% [67]. As mentioned for tensile properties, the influence of the particles’ shape and size on the impact properties have also been assessed; in particular, some researchers have observed that composites with longer fibers provide better impact properties, as there is more distance for the fiber to pull out, and more energy is required [6]. In this study, the biomasses present a very small particle size and a low aspect ratio, which probably counterbalanced the reduced size of the filler, particularly in the case of the unwashed biomass, where the properties were not affected by the amount of filler introduced within the matrix. 

The mechanical behavior of the composites obtained with both the biomasses coincides with the expected one based on the visual appearance of the pieces. The samples obtained with 5% of both the biomasses presented a good appearance and surface quality, and the mechanical properties were not strongly affected in comparison with those of pure PE. However, for higher loadings, the parts obtained with Sp presented a worse appearance, with the presence of some voids in the corners of the cube-shaped pieces and poorer mechanical properties in comparison with those of the Sp.N.W composites. As explained before, this different behavior may be related to the differences in the bulk density of both the biomasses. On the other hand, the presence of salt has been previously related to the enhancement of the mechanical properties of certain bioplastics [38], although no studies correlating the presence of salts with the properties of the composite have been conducted to date in the literature. 

Thus, in general terms, when working with a 5% load, both the biomasses presented similar behaviors. However, for higher loadings, the non-washed Spirulina parts presented a better visual appearance and better mechanical properties, with the added benefit that implies not having to wash the biomass. 

As previously commented, the results obtained in the present study coincide with the behaviors observed in the previously published works on rotomolded composites. In general terms, the mechanical properties are influenced by the introduction of a natural filler into the PE matrix. As the mechanical behavior of the parts shows a direct relationship with the manufacturing process, together with the type of biomass, polymer and additives employed, the results obtained in this manuscript are not directly comparable with the previously reported results about the behavior of microalgae biocomposites, as this is the first work dealing with the use of microalgae in rotational molding, apart from a previous interim work by other authors, in which the processability of *Tetraselmis* by rotomolding was assessed [20]. Just to briefly comment on some of the published works, Otsuki et al. [27] obtained a novel composite of *Chlorella* and PE chemically modified with maleic anhydride by heat pressurizing, and they found that the tensile strength and elongation at break decreased with an increasing biomass content [27]. Similarly, Zhang et al. also reported that the tensile strength of PVC/*Chlorella* composite materials obtained using the heat pressurizing method decreased by increasing the *Chlorella* content, although using less than 20% of load allowed them to meet the requirements for rigid PVC products [26]. 

Due to the different natures of the matrix and the organic fillers, the chemical modification of fillers or the addition of a coupling agent is often performed [13]. This strategy has allowed people to use loads exceeding 10% in rotational molding, where using the upper loads is difficult due to the characteristics of the process [14]. In the case of microalgae composites, most authors have remarked on the need to use a compatibilizer. Various compatibilizers have been used, such as maleic anhydride (MA), grafted ethylene/propylene rubber and diethyl succinate [15]. Among them, MA is one of the most used in microalgae composites. In the conducted studies, the addition of MA increased the homogeneity and flexibility of the products [16]. For example, in a study conducted by Otsuki et al., the tensile strength of a *Chlorella*–MA modified PE composite with a *Chlorella* content of 40 wt % was more than two times greater than that of a composite derived from unmodified PE [27]. In the research conducted by Zhu et al. using maleic anhydride-grafted PBS as a compatibilizer, the polybutylene succinate (PBS)/Spirulina composites presented an improved surface morphology without cracking, reduced pores and better mechanical properties [22]. In other study, *Chlorella vulgaris* and PVA treated with maleic anhydrate allowed the authors to obtain biocomposites with an improved elongation at break, tensile strength and elasticity [34]. Thus, the use of compatibilizers could be a strategy to be explored in the microalgae rotomolded composites field. In any case, the benefits of using compatibilizer agents in rotomolding is still unclear, as pointed in other research works [68,69,70,71]; furthermore, the incorporation of such materials, or the treatment of either the biomass or the polymer, would have a larger environmental impact (and economic cost), which would need to be fully justified by the improvement of the properties obtained. 

#### 3.2.3. Scanning Electron Microscopy (SEM)

**Figure 11:**
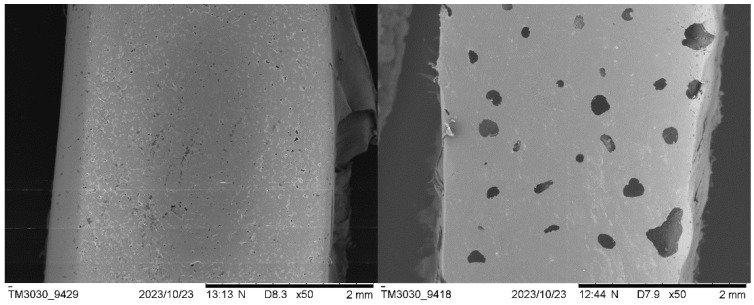
SEM images of external surface of rotomolded parts of 100% PE (**left**) and 10% washed Spirulina (**right**).

The surface of the rotomolded items and the breaking section of the tensile bars were observed using this technology. The SEM images of the surface of neat PE parts (Figure 11) show a homogeneous matrix, where the sintering process was completed and with the presence of very small bubbles that are characteristic of the rotomolding process. In the case of the composite samples, the surface presents a greater amount of larger voids in which the particles of biomass and the PE matrix can be distinguished. 

As expected, the SEM observations correlate with the values of density measured, and the increased porosity occurred with the biomass increase. Although on some occasions, small agglomerations of material can be seen, a good distribution of biomass particles along the matrix was observed in general terms. The fracture surfaces show that the plastic matrix experimented deformation (and particles were not pulled out of the polymeric matrix subjected to plastic deformation, which is indicative of a good interaction) as also observed in the stress–strain diagrams. For a higher biomass content, the capacity of deformation of the matrix is reduced; this result supports the fact that the strain at break decreased as the ratio of biomass increased.

**Figure 12:**
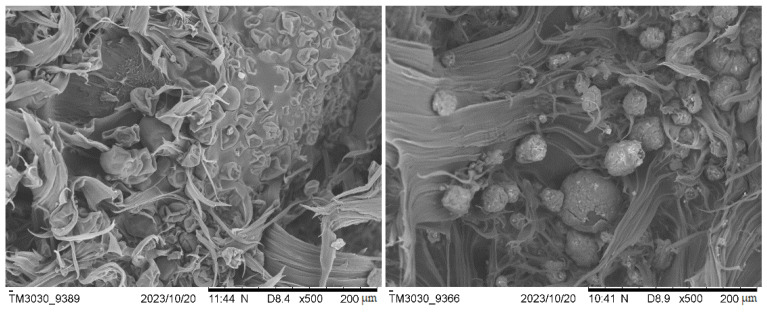
SEM images of fracture surface of tensile specimens composed of a 15% Spirulina (**left**) and 10% non-washed Spirulina (**right**).

Regarding biomass–matrix interaction, it seems that the more irregular shape of the washed Spirulina favors the contact area between the filler and the polymer matrix, improving their bonding. The Sp composites present some areas where the particles of biomass appear embedded in the continuous matrix, while in others, there is lack of adhesion between the matrix and the particles. In the case of the non-washed Spirulina particles, they seem to present a worse interaction due to their spherical shape; in addition, the presence of salt on the surface might also hinder attachment to the polymer. In Figure 12, it can be observed that the particles separated from the matrix, proving that there was poor adhesion. These observations are apparently opposite to the mechanical properties found for the SP.N.W. series, with better mechanical behavior than that of the SP ones. However, this can be explained by the higher density of composites and fewer voids in the washed biomass; therefore, the higher density and lowered porosity of these composites counteract the apparently lower degree of embedding of the unwashed Spirulina within the PE matrix.

If compared with other published works using natural fillers, fewer gaps between the continuous polymer phase and the filler were observed in the present study, which could be related to the high protein content in Spirulina biomass. Hejna et al. concluded that the proteins present in wheat bran could improve the interactions of filler with the matrix [7]. As these materials have a relatively high protein content, this might be acting in such a sense; the presence of salt in the unwashed material made the protein less exposed, hindering such an interaction. 

#### 3.2.4. Thermal Characterization of Composites

**Figure 13:**
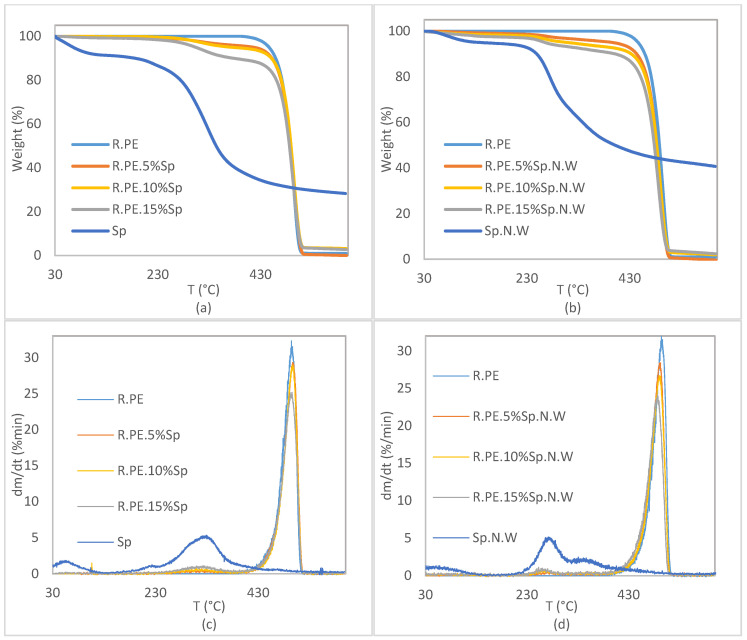
Thermogravimetric (TGA) and derivative (DTG) curves for washed Spirulina (**a**,**c**) and non-washed Spirulina (**b**,**d**) composite materials.

TGA and DTG curves obtained from the thermogravimetric analysis of the composite materials are represented in Figure 13. It can be observed that the degradation of pure PE is a one-step process, while the composite curves show two degradation steps, corresponding the first small step to the thermal degradation of Spirulina and non-washed Spirulina and the second one to polymer thermal decomposition. The addition of the biomass resulted in a reduction in the thermal stability of the composite, as was expected.

The temperatures at which the 5% (T_5%_) 10% (T_10%_), and 50% (T_50%_) mass loss occurred, the DTG peak values, and the residual mass are included in Table 5. Although in the case of the Sp.N.W composites, a very slight reduction in the DTG peak value (corresponding to PE degradation) was observed, in general, it can be concluded that the addition of a filler did not affect to the thermal behavior of the matrix itself. If the values of T_5%_ are compared, it can be seen that its value was considerably reduced, with this reduction being higher for the Sp.N.W composites. For maximum biomass loading, reductions of 32% and 41% in the T_5%_ values were obtained for R.PE.15%Sp and R.PE.15%Sp.N.W, respectively, compared with that of neat PE. Even although, the T_5%_ values significantly exceed the mold temperature used for composite production, and therefore the observed adverse changes will not affect the processability of the material and will not deteriorate the application characteristics of the final rotomolded parts.

Additionally, differential scanning calorimetry analyses were conducted, in which no significant changes in the thermal properties (melting and crystallization temperatures) were observed (Table 6). DSC graphs are included in the Supporting Information (Figure S1). In some cases, the peak that is supposed to correspond to protein denaturation appears after the PE melting peak observed during the first heating. However, in other cases, this peak was not observed. The plasticization of some part of the protein present in the biomass could occur during rotomolding, and the small amount of material that is used during DSC analysis and the non-homogeneity of the composites could explain the arbitrariness in the observation of protein denaturation peak in the analysis of the composites. 

The oxidation induction time (OIT) was also determined using DSC analysis as an indicator of the effective antioxidant level present in the test specimen. The results are included in Table 6. As can be seen, the OIT values were increased compared with those of PE, which points to the important antioxidant activity of microalgae related to their content of antioxidant compounds. Polyphenols contents of 367.4 mg/100 g and 108.34 mg/100 g in the dry biomass measured using the Folin–Ciocalteu method were obtained for the Sp and Sp.N.W biomasses, respectively. This difference in the concentration of antioxidants is reflected in the antioxidant effect obtained as composites produced with the washed biomass reached oxidation later, and even for 15% of Spirulina, the event was not well defined.

It should be noted that the OIT tests in this study were performed in an air atmosphere instead of oxygen, as used in many other works. In addition, the other setting conditions used during the test affected the time at which the oxidation happened, which is why the values of oxidation induction time are not directly comparable with the other published results, although they can be compared with those of the pure polymer. For example, Hejna et al. found that coffee silverskin addition (20 wt%) increased the oxidation induction of HDPE in 120 min [45]. In a different study, Hejna et al. observed that the addition of coffee and cocoa industry by-products (20% of load) extended the oxidation induction time by 100% (54 min) compared to that of pure PE (20 min) [46]. Different authors have shown the relevance that biomass can have in obtaining functionalized composites based on polymer matrices, particularly when taking advantage of the high antioxidant content in such materials. This is pointed out as an interesting alternative to replace synthetic stabilizers, the use of which had been restricted in recent years [9,45,72,73]. On this occasion, the temperature and air flow used during the test made sure that pure PE oxidation started from the beginning of the assay (time 0.0 min). The test conditions could not be changed, as otherwise, the oxidation event would not have been observable for all the composites, but in any case, the beneficial effect of biomass addition on the thermooxidative resistance of the composites was evident.

#### 3.2.5. FTIR

**Figure 14:**
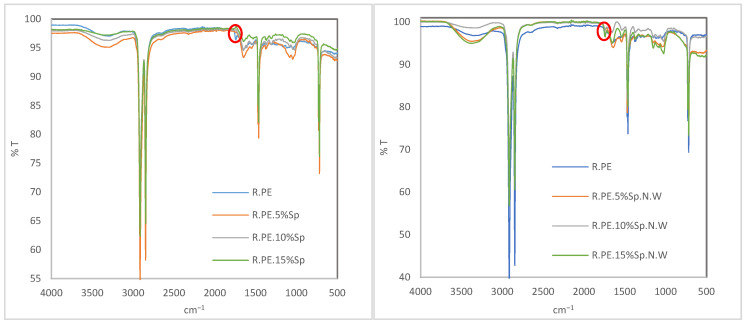
FTIR spectra of washed Spirulina (**left**) and non-washed Spirulina (**right**) composite materials.

In Figure 14, the FTIR spectra of the rotomolded composites are presented. All the samples showed strong bands corresponding to the PE characteristic bands and other softer bands corresponding to the biomass. If special attention is paid to the range between 1650 and 1800 cm^−1^, the range where the influence of oxidation can be evaluated [13], a different behavior is observed for both the biomasses. There is a peak at around 1714 cm^−1^ (marked in red in Figure 14) in the pure PE samples and composites obtained with Sp.N.W, but not in the case of the Sp composites [74,75]. This peak may be correlated with carbonyl compounds formed during the oxidation of polyethylene, which could suggest that Spirulina biomass could have had an antioxidant effect. This is in agreement with the OIT results, in which Spirulina provides more stability than unwashed biomass. In any case, the relative relevance of this peak for unwashed Spirulina is lower than that for PE, somehow also indicating a stabilizing action against the oxidation of PE during processing.

#### 3.2.6. Water Absorption

Due to the use of composite materials in a wide range of industries, such as decks, floors and outdoor facilities, it is necessary to analyze the water absorption behavior of natural composite fillers [76]. This is an important property for composites obtained with natural fillers as their introduction affects the hygroscopic character of the material, which ultimately will have an effect on their mechanical properties [77]. The introduction of a filler in a composite material will affect the porosity of the material, and the voids in its structure will compromise its mechanical properties not only because of their presence, but also because they increase the absorption of water [78]. This is especially important in rotomolding, where due to the characteristics of the process in which pressure is not used, high porosities are obtained. On the other hand, not only the existence of voids, but also the chemical affinity and type of matrix, temperature, polarity, diffusivity, and hydrogen bond formation, in addition to the nature, volumetric fraction, orientation, porosity, and geometry of fillers will affect the water absorption of the composites [79,80]. 

In this study, the water absorption behavior of this microalgae composite was studied. On the other hand, due to the capacity of the solubilization of microalgae biomass in water, not only the water absorption, but also composite solubilization (which was taken into consideration for absorption correction) during the time was evaluated. Table 7 presents the water absorption uptake at equilibrium (no changes were observed after 40 days of assay). For the pure PE samples, water absorption was almost negligible. However, as expected, water absorption increased with the percentage of filler for both the biomasses, which correlates with porosity; the composites with higher porosity exhibit more water uptake due them having to a larger number of entry points where the water molecules can access. This is an expected behavior previously commented in the literature for composites with biomass, which are generally hydrophilic; under constant environmental conditions, the moisture content tends to increase with the volumetric fraction of fillers in the composites, particularly for vegetal-derived fillers [78], and property losses have been observed for polymeric composites reinforced by plant fibers of a different nature, such as reed [81], bamboo [82], flax [83], hemp [84] and jute [85].

Similar values of solubilization were obtained for both the biomasses, but higher absorption values were obtained for the Sp composites, which is also in accordance with the higher quantity of voids observed. The value of moisture uptake obtained at equilibrium (18.8%) is comparable with the one obtained by other authors working with natural fillers in rotational molding. For example, water absorption values up to 17.4% and 21.1% were obtained with the incorporation of a 20% agave and 30% coir fibers, respectively [86]. In the same study, they got to reduce these values, improving interfacial compatibility, via the surface treatment of the fibers. Although high values of water absorption were obtained in the present study, the values are still lower than 25%, which is considered as the minimum level necessary to start affecting the mechanical properties and causing bacterial growth [87]. Therefore, even the maximum water uptake obtained in this study, which was 18.7% for the R.PE.15%Sp composite, guarantees dimensional stability and no growth of bacteria and fungi.

## 4. Conclusions

In this paper, Spirulina biomass/PE biocomposite materials were obtained by rotational molding. The influence of the biomass percentage in the mechanical, thermal and water absorption behaviors of the composites obtained was evaluated. A comparison of the use of washed and non-washed Spirulina biomasses was also conducted. Composites with up to 15% loads were satisfactory processed with both the biomasses, showing a good aesthetic appearance with good blending and distribution of the filler in the matrix. The composites with 5% of both the biomasses did not significantly modify their mechanical behavior when compared with those of the neat PE. For higher loadings, in general, all the mechanical properties were decrease by the incorporation of both fillers, although they were less affected in the case of non-washed Spirulina; the non-washed Spirulina parts presented a better visual appearance and improved mechanical properties, with the added benefit that implies not having to wash the biomass. On the other hand, although the addition of the biomass resulted in a reduction in the composites’ thermal stability, the observed adverse changes did not affect to the processability of the material, as the rotational molding process was performed at temperatures below those of biomass degradation. Moreover, in relation to the values of water absorption obtained, even the maximum water uptake obtained in this study guarantees dimensional stability and no growth of bacteria and fungi. Finally, especially interesting was the capacity of the biomass to prevent the thermooxidation of polyethylene. The oxidation induction time values were notably increased when adding the biomass, with this being more evident for the washed biomass composites. This constitutes a novel field of research, in which a biomass is introduced as a filler of composites materials not only to reduce the potential cost of the products or to potentially improve their sustainable character, but also as a way to provide novel features, i.e., increased stability against thermooxidation in the substitution of compounds of synthetic origin. The implementation of new strategies for the valorization of such resources with a smaller environmental impact (such as in the case of unwashed Spirulina) contributes to the achievement of new functional materials with improved behavior, particularly tailored to the rotomolding sector.

## Figures and Tables

**Table 1:** Composite materials obtained by rotational molding.

Biomass	Specimen	% Biomass
	R.PE	0
Spirulina	R.PE.5%Sp	5
	R.PE.10%Sp	10
	R.PE.15%Sp	15
Spirulina non-washed	R.PE.5%Sp.N.W	5
	R.PE.10%Sp.N.W	10
	R.PE.15%Sp.N.W	15

**Table 2:** Degradation temperatures (°C) and weight loss (%) obtained in the thermogravimetric analysis of Spirulina and non-washed Spirulina.

Microalgae	T Range (°C)	% Weight Loss	T Peak (°C)
Spirulina	30–140	9.26 ± 1.12	60
	140–240	5.73 ± 0.12	228
	240–600	56.87 ± 1.37	330
Non-washed Spirulina	30–150	7.90 ± 2.46	60
	150–320	29.40 ± 1.19	274
	320–600	22.25 ± 1.13	342

**Table 3:** Average values (±standard deviations) for mechanical properties of materials.

Specimen			Flexural Test		Tensile Test	
	Density (g/cm^3^)	Impact Strength (kJ/m^2^)	Flexural Strength (MPa)	Flexural Elastic Modulus (MPa)	Tensile Strength (MPa)	Tensile Elastic Modulus (MPa)
R.PE	0.892 ± 0.002	17.19 ± 2.72 ^a^	16.61 ± 0.94 ^a^	582.85 ± 20.15 ^a^	15.71 ± 0.54 ^a^	571.03 ± 40.65 ^a^
R.PE.5%Sp	0.852 ± 0.009	14.07 ± 1.56 ^a^	14.38 ± 1.21 ^a^	520.65 ± 49.10 ^a^	12.01 ± 0.30 ^ab^	452.83 ± 50.03 ^ab^
R.PE.10%Sp	0.807 ± 0.012	9.08 ± 1.73 ^ab^	9.91 ± 1.01 ^ab^	380.49 ± 55.78 ^ab^	8.40 ± 0.30 ^bc^	447.57 ± 37.66 ^ab^
R.PE.15%Sp	0.724 ± 0.018	5.34 ± 1.74 ^b^	6.32 ± 0.75 ^b^	253.44 ± 49.41 ^b^	4.61 ± 0.35 ^c^	315.01 ± 28.24 ^b^
R.PE	0.892 ± 0.002	17.19 ± 2.72 ^a^	16.61 ± 0.94 ^a^	582.85 ± 20.15 ^a^	15.71 ± 0.54 ^a^	571.03 ± 40.65 ^a^
R.PE.5%Sp.N.W	0.890 ± 0.002	11.38 ± 2.68 ^ab^	12.47 ± 0.69 ^ab^	492.41 ± 26.15 ^ab^	12.85 ± 0.45 ^ab^	598.58 ± 39.22 ^a^
R.PE.10%Sp.N.W	0.888 ± 0.003	12.67 ± 2.45 ^ab^	12.19 ± 0.65 ^ab^	501.59 ± 36.64 ^ab^	11.97 ± 0.51 ^bc^	587.71 ± 43.14 ^a^
R.PE.15%Sp.N.W	0.878 ± 0.011	11.81 ± 1.75 ^b^	9.87 ± 0.57 ^b^	419.92 ± 38.81 ^b^	10.45 ± 0.55 ^c^	542.04 ± 50.45 ^a^

Tukey–Kramer test was used for the comparison of properties of the different series of materials. Those materials with the same superscript letter show no statistical difference (at 95% confident level) for the property.

**Table 4:** The coefficient of determination (R^2^) and the estimated regression coefficients of the equation of first order (property = a + b × %biomass) obtained from the adjustment of the experimental values of the mechanical properties of the Sp composites.

Property	a	b	R^2^
Tensile strength	15.72	−0.739	0.999
Tensile elastic modulus	562.61	−15.47	0.911
Flexural strength	17.109	−0.707	0.985
Flexural elastic modulus	603.02	−22.517	0.977
Impact strength	17.502	−0.8104	0.993

**Table 5:** The temperatures at which the 5% (T_5%_), 10% (T_10%_) and 50% (T_50%_) mass loss occurred, the DTG peak values, and the residual mass obtained in the thermogravimetric analysis of the Sp and Sp.N.W composite materials.

Specimen	T_5%_ (°C)	T_10%_ (°C)	T_50%_ (°C)	DTG Peak [%/min; °C]	Residual Mass (%)
R.PE	446.07	459.17	487.52	32.31; 494.64	0.92
R.PE.5%Sp	415.82	451.58	488.67	29.32; 497.18	0.00
R.PE.10%Sp	372.82	450.78	489.05	28.96; 497.03	3.17
R.PE.15%Sp	303.30	375.92	485.91	25.15; 495.93	2.70
R.PE.5%Sp.N.W	397.11	443.24	482.48	28.42; 492.63	0.00
R.PE.10%Sp.N.W	309.71	432.85	482.32	26.70; 490.96	1.94
R.PE.15%Sp.N.W	264.99	402.08	477.47	24.10; 486.78	2.36

**Table 6:** Thermal parameters obtained from DSC analysis of composites (Tm_1_—melting temperature 1st heating; Tm_2_—melting temperature 2nd heating; Tc—crystallization temperature; Xc—crystallinity, OIT—oxidation induction time).

Specimen	Tm1	Tc	Tm2	Xc	OIT (min)
R.PE	128.56	108.92	127.55	35.99	0.0
R.PE.5%Sp	127.86	109.64	126.88	42.11	27.9
R.PE.10%Sp	130.64	109.38	127.26	41.03	58.0
R.PE.15%Sp	127.97	109.62	126.70	37.40	0.0
R.PE.5%Sp.N.W	128.58	109.26	127.41	41.72	14.6
R.PE.10%Sp.N.W	128.73	109.19	127.42	30.06	27.8
R.PE.15%Sp.N.W	128.80	109.68	126.96	40.07	41.5

**Table 7:** Water absorption (after solubilization correction) and solubilization values of composite materials after 60 days of testing.

Specimen	% W	% Solubilization
R.PE	0.34 ± 0.02	-
R.PE.5%Sp	3.63 ± 0.28	0.42 ± 0.04
R.PE.10%Sp	11.68 ± 0.20	1.01 ± 0.71
R.PE.15%Sp	18.77 ± 1.29	4.50 ± 0.99
R.PE.5%Sp.N.W	2.99 ± 0.43	0.72 ± 0.16
R.PE.10%Sp.N.W	4.49 ± 0.39	1.71 ± 0.18
R.PE.15%Sp.N.W	10.40 ± 1.38	4.63 ± 1.81

## Data Availability

The original contributions presented in the study are included in the article/Supplementary Materials, further inquiries can be directed to the corresponding authors.

## Supplementary Materials

The following supporting information can be downloaded at: https://www.mdpi.com/article/10.3390/polym16131807/s1, Figure S1. DSC curves for washed Spirulina (left) and non-washed Spirulina (right) composite materials, Table S1. Surface elemental composition of Spirulina and Spirulina non-washed biomasses obtained using SEM-EDX analytical system.
