# Supplementary material for: Characterization of Microalgae Biomass-Based Composites Obtained through Rotational Molding
Source: Polymers (Basel). 2024 Jun 26;16(13):1807. doi: 10.3390/polym16131807 (PMC11243890; doi:10.3390/polym16131807)
Supplement: Supplementary file 1 [file polymers-16-01807-s001.zip › polymers-3053764-supplementary.pdf]

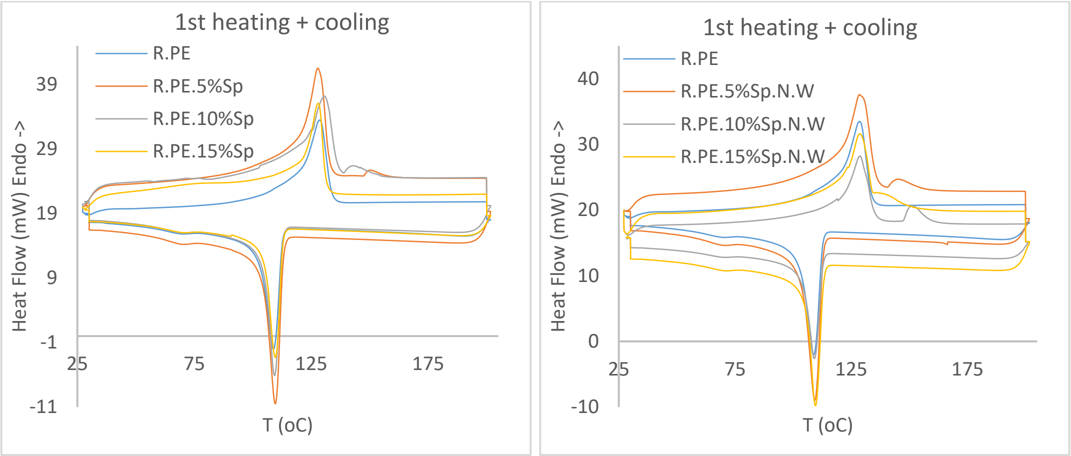

Figure S1. DSC curves for washed *Spirulina* (left) and non-washed *Spirulina* (right) composite materials

Table S1. Surface elemental composition of Spirulina and Spirulina non-washed biomasses obtained with SEM-EDX analytical system

**Element**	**wt (%)**	
	Spirulina	*Spirulina* non-washed
Cl	20.97	47.50
P	20.30	4.34
K	19.72	9.63
S	12.93	6.11
Mg	9.73	7.21
Na	9.42	22.80
Ca	6.72	2.13
Si	0.22	0.27
N	0.00	0.00
